# Osteoclasts on Bone and Dentin In Vitro: Mechanism of Trail Formation and Comparison of Resorption Behavior

**DOI:** 10.1007/s00223-013-9786-7

**Published:** 2013-09-11

**Authors:** M. Rumpler, T. Würger, P. Roschger, E. Zwettler, I. Sturmlechner, P. Altmann, P. Fratzl, M. J. Rogers, K. Klaushofer

**Affiliations:** 1 1st Medical Department, Ludwig Boltzmann Institute of Osteology at the Hanusch Hospital of WGKK and AUVA Trauma Center, Hanusch-Krankenhaus, Pav. III, UG, Heinrich Collin Strasse 30, 1140 Vienna, Austria; 2 Department of Biomaterials, Max Planck Institute of Colloids and Interfaces, Potsdam, Germany; 3 The Garvan Institute of Medical Research, Darlinghurst, NSW Australia

**Keywords:** Osteoclast, Bone, Dentin, Resorption model, Trail formation

## Abstract

The main function of osteoclasts in vivo is the resorption of bone matrix, leaving behind typical resorption traces consisting of pits and trails. The mechanism of pit formation is well described, but less is known about trail formation. Pit-forming osteoclasts possess round actin rings. In this study we show that trail-forming osteoclasts have crescent-shaped actin rings and provide a model that describes the detailed mechanism. To generate a trail, the actin ring of the resorption organelle attaches with one side outside the existing trail margin. The other side of the ring attaches to the wall inside the trail, thus sealing that narrow part to be resorbed next (3–21 μm). This 3D configuration allows vertical resorption layer-by-layer from the surface to a depth in combination with horizontal cell movement. Thus, trails are not just traces of a horizontal translation of osteoclasts during resorption. Additionally, we compared osteoclastic resorption on bone and dentin since the latter is the most frequently used in vitro model and data are extrapolated to bone. Histomorphometric analyses revealed a material-dependent effect reflected by an 11-fold higher resorption area and a sevenfold higher number of pits per square centimeter on dentin compared to bone. An important material-independent aspect was reflected by comparable mean pit area (μm^2^) and podosome patterns. Hence, dentin promotes the generation of resorbing osteoclasts, but once resorption has started, it proceeds independently of material properties. Thus, dentin is a suitable model substrate for data acquisition as long as osteoclast generation is not part of the analyses.

## Introduction

The main physiological function of osteoclasts in vivo is the resorption of bone matrix, which precedes the formation of new bone; this skeletal remodeling cycle allows adaptation to physical loading conditions, which in turn maintains the integrity of the skeleton [1]. This cycle also exhibits a key function in skeletal repair, which is a fundamental process ranging from the healing of traumatic fractures to the healing of osteotomies performed during reconstructive surgery [2]. An imbalance in osteoclastic activity can lead to implant loosening or bone diseases. Increased resorption can lead to postmenopausal osteoporosis, whereas dysfunction of osteoclasts leads to osteopetrosis [3].

Resorbing osteoclasts develop by the fusion of mononuclear precursor cells of the monocyte–macrophage lineage in the presence of the osteogenetic cytokines macrophage-colony stimulating factor (M-CSF) and receptor activator of nuclear factor-κB ligand (RANKL) [4]. In order to resorb bone, osteoclasts become polarized, which involves the organization of the F-actin cytoskeleton into a typical densely packed ring-like structure, thus sealing the area beneath and forming a closed compartment in which the bone matrix and mineral can be resorbed, resulting in the formation of resorption pits [5, 6]. The sealing zone is composed of an F-actin ring, consisting of podosomes that mediate the attachment of the cells to the extracellular matrix and encircling a highly ruffled secretory membrane [6, 7]. Luxenburg et al. [8] demonstrated that during the polarization process new actin is recruited to fibers that directly interconnect the podosome actin cores and undergo dramatic reorganization during osteoclast maturation. During the resorption process, bone mineral, mainly hydroxyapatite, is dissolved by the secretion of acid that is brought about by fusion of acidic vesicles to the plasma membrane and insertion of proton pumps into the ruffled border membrane. The organic matrix is degraded by proteolytic enzymes, e.g., matrix metalloproteinases and cathepsin K, that are released into the resorption lacunae with further digestion/transport of internalized protein fragments. Nevertheless, this general task can be regulated by a variety of factors including the chemical and physical nature of the mineralized resorption substrate. The process of osteoclastic activity leaves behind resorption traces in the form of pits or trails, which are generally used to describe osteoclastic resorption behavior. Formation of a single resorption pit occurs when an osteoclast resorbs a discrete cavity beneath the cell [9], but the formation of resorption trails is less well understood.

The formation of pits and trails is a general hallmark of osteoclastic activity, observed in pure cultures or in coculture with other cell types from a variety of tissue sources (bone marrow, bone, blood, or cell lines) and species (human, mouse, rat, rabbit). Additionally, the same pits or trails are generated regardless of the offered mineral substrate (dentin, bone, artificial ceramic, or hydroxyapatite substrates). For in vitro investigations, dentin is often used as a mineralized substrate, and data obtained from such experiments are often extrapolated to osteoclastic behavior on bone [10–13]; but there is little information regarding direct comparison between these two materials, bone and dentin.

For this reason, we generated human osteoclasts from cultured precursor cells isolated from human peripheral blood and analyzed and compared the resorption behavior of these osteoclasts on these two materials histomorphometrically. Beyond that, we studied trail-forming osteoclasts during the resorption process with confocal laser scanning microscopy and provide a model of how resorption trails occur.

## Materials and Methods

### Antibodies and Chemicals

Primary antibodies included monoclonal anti-vinculin (clone hVIN-1; Sigma, St. Louis, MO), used in dilution 1:250; monoclonal anti-vitronectin receptor (CD51/CD61; AbD Serotech, Düsseldorf, Germany), used in dilution 1:1,000; and monoclonal anti-paxillin (BD Transduction Laboratories, Lexington, KY), used in dilution 1:250. Secondary antibodies included Alexa Fluor 488 goat anti-rabbit IgG (Invitrogen, Life Technologies, Carlsbad, CA), used in dilution 1:250; phalloidin-TRITC (Sigma), used at a concentration of 4 × 10^−8^ M; and Western blocking reagent (Roche, Indianapolis, IN). Dentin of elephant tusk (ivory) was kindly provided by German Customs in accordance with international laws.

### Mineralized Tissue Substrates

As a model system, we chose two different mineralized substrates, bone (bovine cortical bone) and dentin (elephant ivory), which are widely described in the literature. The bone was stored at −20 °C until use. Pieces with a thickness of approximately 300 μm were cut with a diamond saw. Slices directly obtained from sawing were used as rough substrates and slices that had been polished with tissue loaded with diamond grains were used as smooth substrates. Slices were cleaned by ultrasonication in distilled water and placed in 70 % ethanol for 5 min for sterilization before use.

### Profilometry

The surface roughness of the bone and dentin slices was determined by profilometry at the Materials Center Leoben (Leoben, Austria) using a Nanofocus μsurf Confocal Microscope (Nanofocus, Oberhausen, Germany). Values were obtained over a scan length of approximately 600–1,300 μm to gain *R*_a_ and *R*_q_. *R*_a_ is the arithmetic average of the absolute values of all points of the profile, also called “average roughness value”; *R*_q_ is the root mean square of the values of all points of the profile.

**Table:** 

	Bone rough	Bone smooth	Dentin rough	Dentin smooth
*R*_a_ (μm)	6.27	1.05	15.14	3.75
*R*_q_ (μm)	7.71	2.27	18.51	5.21

### Osteoclast Cultures

Osteoclasts were generated from human peripheral blood mononuclear cells obtained from 16 healthy donors (aged 19–55 years) as previously described [14]. Experiments were approved by the Austrian Ethics Committee, and informed consent was obtained from all volunteers. Briefly, peripheral blood mononuclear cells were isolated by centrifugation over a Lymphoprep gradient and then seeded into a Petri dish containing αMEM (Sigma) supplemented with 10 % fetal calf serum (FCS; Thermo Fisher, Geel, Belgium), 2 mM l-glutamine, 20 ng/mL M-CSF (R&D Systems, Wiesbaden-Nordenstadt, Germany), and 30 μg/mL gentamycin. This resulted in a pure preosteoclastic cell culture with no contamination by other cell populations. After approximately 10 days in culture (preculture), adherent cells were removed using trypsin and experiments were seeded into 48-well plates containing bone or dentin slices at a density of 7.2 × 10^4^ cells/cm² in αMEM supplemented with 10 % FCS, 2 mM l-glutamine, 30 μg/mL gentamycin, 20 ng/mL M-CSF, and 2 ng/mL RANKL (R&D Systems, Wiesbaden-Nordenstadt, Germany). Culture medium was changed twice per week.

### “Resorbing” and “Nonresorbing” Osteoclast Cultures

To generate the two populations (1) “resorbing osteoclasts” and (2) “nonresorbing osteoclasts,” we isolated osteoclasts from human peripheral blood mononuclear cells from two different donors as described in “Osteoclast Cultures.” The resorption activity of the osteoclasts from these two donors was known from previous experiments as a resorbing or nonresorbing population. These cells were also introduced into a 10-day preculture period in αMEM supplemented with 10 % FCS, 2 mM l-glutamine, 20 ng/mL M-CSF, and 30 μg/mL gentamycin. Afterward, cells were seeded into 48-well plates containing bone or dentin slices at a density of 7.2 × 10^4^ cells/cm² in αMEM supplemented with 10 % FCS, 2 mM l-glutamine, 30 μg/mL gentamycin, 20 ng/mL M-CSF, and 2 ng/mL RANKL. These cultures are referred to here as “resorbing” and “nonresorbing” osteoclasts. No additional substances were added to influence resorption activity in those two populations. Culture medium was changed twice per week.

### Cell Adhesion on Mineralized Substrates

For cell adhesion studies, after 10 days of preculture, cells were seeded onto bone or dentin slices (as described above) in αMEM supplemented with 10 % FCS, 2 mM l-glutamine, 30 μg/mL gentamycin, 20 ng/mL M-CSF, and 2 ng/mL RANKL and fixed 6 h after seeding in a solution of 4 % paraformaldehyde in PBS for 20 min. After staining with 1 % crystal violet for 20 min at room temperature, cells were washed extensively with PBS and the number of cells adhered to the surfaces was determined under a reflected light microscope. The number of cells was then normalized to the area of substrate and expressed as cells per square centimeter. Statistical analysis was carried out using a *t* test (Prism 4.0; GraphPad Software, San Diego, CA).

### Histomorphometric Measurements/Assessment of Osteoclastic Resorption

After 10 days of preculture in the medium described above, cells were seeded onto bone and dentin slices at a density of 7.2 × 10^4^ cells/cm² in αMEM supplemented with 10 % FCS, 2 mM l-glutamine, 30 μg/mL gentamycin, 20 ng/mL M-CSF, and 2 ng/mL RANKL and kept in culture for 14 days. After this culture time, substrates were put into water, sonicated for 10 min to remove cells, and air-dried. Photographs were obtained by reflected light microscopy (objective 20×) of the entire substrate surface, and resorption trails were analyzed with standard image analysis software (ImageJ, rsbweb.nih.gov/ij/). For quantitative analysis, pit areas were defined by an area of resorption surrounded by a margin of unresorbed material. We counted the number of “resorption events” on the substrates and measured their area, length, and width to obtain the following parameters: resorbed area (percent), mean pit size (square micrometers), number of pits per square centimeter, and mean pit length and width (micrometers). We did not distinguish between pits and trails in these analyses. Statistical analysis was done by *t*-test (Prism 4.0), and data were represented as mean ± SEM.

### Immunofluorescence

For cell adhesion studies, after 10 days of preculture, cells were seeded onto bone or dentin substrates in αMEM supplemented with 10 % FCS, 2 mM l-glutamine, 30 μg/mL gentamycin, 20 ng/mL M-CSF, and 2 ng/mL RANKL. After 8 h, cells were fixed in a solution of 4 % paraformaldehyde in PBS for 20 min, washed with PBS, and permeabilized in 0.1 % Triton X-100. After blocking with 10 % Western blocking reagent (Roche) for 20 min at room temperature, cells were incubated with the primary antibody (anti-vinculin or anti-paxillin) diluted in 10 % Western blocking reagent for 1 h. After several washing steps with PBS, cells were incubated with an Alexa Fluor 488–labeled goat anti-mouse IgG antibody for 1 h. Afterward, the actin cytoskeleton of the cells was stained with TRITC-labeled phalloidin for 40 min and the mineralized substrate was stained with 10 μg/mL calcein for 30 min. Substrates were mounted on glass slides and examined using a confocal laser scanning microscope (TCS SP5; Leica, Solms, Germany).

For determination of pit volume and depth, cells were removed from the substrate surface and the mineralized matrix of the substrate was stained with 10 μg/mL calcein for 30 min, followed by washing with PBS and air drying. *Z*-stacks of each resorption pit were obtained with a confocal laser scanning microscope (Leica TCS SP5), followed by image analysis and volume calculations. Pit depth was determined by multiplication of the number of *z*-slices with step size. Two independent experiments, obtained from two blood samples, were done from each donor.

For 3D analyses of resorbing osteoclasts, after 10 days of preculture, cells were seeded onto bone or dentin substrates in αMEM supplemented with 10 % FCS, 2 mM l-glutamine, 30 μg/mL gentamycin, 20 ng/mL M-CSF, and 2 ng/mL RANKL. After 14 days, cells were fixed with 4 % paraformaldehyde in PBS for 20 min and washed with PBS. After blocking with 10 % Western blocking reagent for 20 min at room temperature, cells were incubated with anti-VNR antibody in 5 % Western blocking reagent for 1 h, followed by incubation with an Alexa Fluor 633-labeled goat anti-mouse IgG antibody for 1 h. After washing with PBS, the actin cytoskeleton of the cells was stained with TRITC-labeled phalloidin for 40 min. Then the sample was stained with 10 μg/mL calcein, washed with PBS, and examined under the confocal laser scanning microscope. One representative resorbing osteoclast is shown.

The horizontal distances between the two sides of typical crescent-shaped actin rings were measured on 2D images for 65 single resorbing cells from pictures obtained under a fluorescence microscope (Eclipse 80i; Nikon, Tokyo, Japan).

### Affymetrix GeneChip Analysis and qRT-PCR

Total RNA was isolated from osteoclasts seeded on bone and dentin (after a 10-day preculture, as described above) after 14 days of culture in αMEM supplemented with 10 % FCS, 2 mM l-glutamine, 30 μg/mL gentamycin, 20 ng/mL M-CSF, and 2 ng/mL RANKL using a RNA Isolation Kit (Promega, Southampton, UK). The RNA for microarray analyses of resorbing osteoclasts was isolated from those cultures which showed good resorption on bone and dentin substrates (verified by histomorphometry). RNA for microarray analyses of nonresorbing osteoclasts was isolated from those cultures which showed no resorption on bone and dentin substrates. For GeneChip analysis, processing of the RNA, such as quality control, cDNA amplification, labeling, hybridization, and scanning of the hybridized arrays, was performed on an Affymetrix Human Gene 1.0 ST Array by the Kompetenzzentrum für Fluoreszente Bioanalytik (Regensburg, Germany). A signal ratio beyond 1.3-fold indicates gene upregulation [15]. For qRT-PCR, cDNA synthesis was performed on 1 μg RNA using the 1st Strand cDNA Synthesis Kit as described by the supplier (Roche). The cDNA was then introduced into PCR amplification using FastStart SYBR-Green Master Mix on a real-time cycler (Rotorgene; Qiagen, Hilden, Germany). PCRs were performed according to the following cycling program: 10 min of initial denaturation at 95 °C, followed by 60 cycles of 25 s denaturation at 95 °C, 45-second annealing at 62 °C, and 30-second extension at 72 °C.

Primer sequences were as follows: cathepsin K (forward) 5′-TGAGGCTTCTCTTGGTGTCCATAC-3′, (reverse) 5′-AAAGGGTGTCATTACTGCGGG-3′; RANK (forward) 5′-GCTCCTCCATGTACCAGTGAG-3′, (reverse) 5′-ACTGTCAGAGGTAGTAGTGCATT-3′; TRAP (forward) 5′- GACCACCTTGGCAATGTCTCTG-3′, (reverse) 5′- TGGCTGAGGAAGTCATCTGAGTTG-3′.

All PCRs were performed in triplicate. Gene expression was normalized to 18Sr RNA.

Statistical analysis was done by *t* test (Prism 4.0), and data are represented as mean ± SE.

## Results

### Cell Adhesion on Bone and Dentin

To verify the adhesion of preosteoclasts on bone and dentin substrates in our culture system we visualized podosome patterns using immunofluorescence-labeled antibodies against two known and well-defined focal adhesion-associated molecules, vinculin and paxillin.

**Fig. 1:**
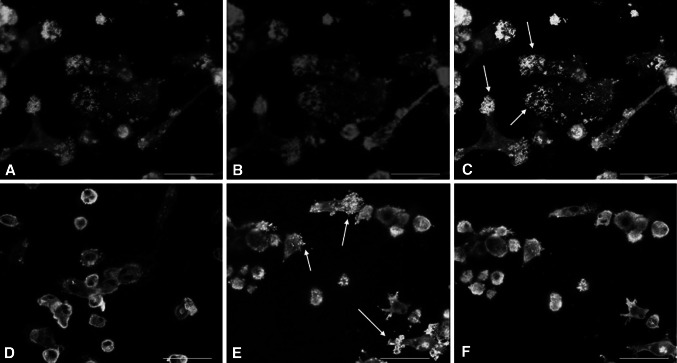
Immunofluorescence for vincullin and actin on preosteoclasts on bone and dentin. Signals: actin (*red*), vinculin (*green*), colocalization of vinculin and actin gives a brilliant yellow color (*white arrows*). **a** Actin staining of cells on dentin surface; image plane: cell attachment site to matrix. **b** Vinculin staining of cells on dentin surface; imaged plane: cell attachment site to matrix. **c** Merged image of **a** and **b**. **d** Merged image of actin and vinculin staining of cells seeded on dentin; image plane: middle of the cell body. **e** Merged image of actin and vinculin immunofluorescence of cells seeded on bone; image plane: cell attachment site to matrix. **f** Merged image of actin and vinculin staining of cells seeded on bone; image plane: middle of the cell body. At attachment sites of cells to the substrate, vinculin and actin show a perfect colocalization in the dot-like adhesion sites (podosomes) on dentin as well as on bone. This colocalization is lost in the cell body on both mineralized materials. *Scale bar* 50 μm

At the contact site between cells and substrate, vinculin was located mainly in the dot-like adhesion structures (podosomes) on dentin as well as on bone and showed a perfect colocalization with actin (Fig. 1c, e). In the main cell body this colocalization was lost and vinculin showed a strong accumulation in the cytoplasm, where as actin was localized mainly at the cell periphery (Fig. 1d, f). The same protein distribution pattern was observed for paxillin when cells were cultured on dentin or bone (data not shown). Hence, we could not find a difference in the appearance of the dot-like cell adhesion patterns of podosomes between bone and dentin concerning those two adhesion molecules.

**Fig. 2:**
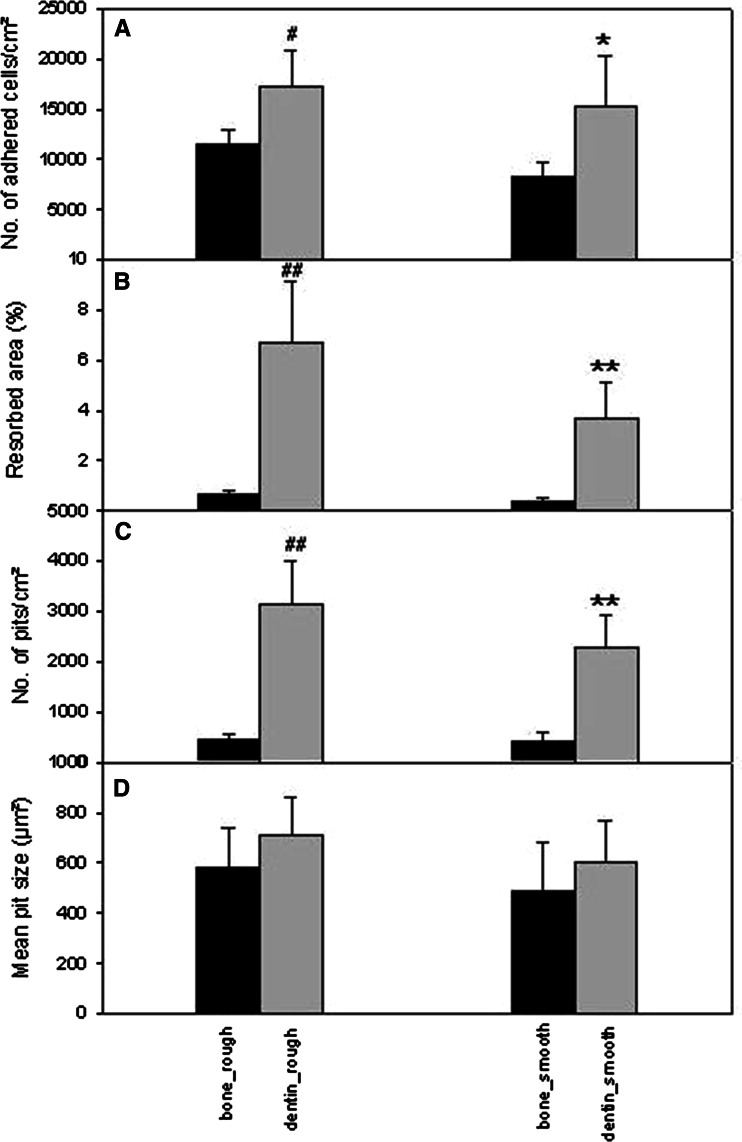
Histomorphometric analyses of osteoclastic resorption on bone and dentin, on rough and smooth surfaces, respectively. **a** Number of cells adhered on the surface 6 h after seeding. *n* = 6 different donors. ^#^ *p* < 0.05; *p < 0.05. **b** Total resorbed areas (%). ^##^ *p* < 0.01; **p < 0.01. **c** Number of pits per square centimeter. ^##^ *p* < 0.01; **p < 0.01. **d** Mean pit size. In case of **b**–**d**: *n* = osteoclasts from 16 different donors. *Bars* represent mean ± SEM; *hash* represents dentin_rough versus bone_rough; *asterix* represents dentin_smooth versus bone_smooth

Cell adhesion on both materials was quantified and revealed approximately 11,400 adhered cells/cm² in case of bone and 17,200 cells/cm² in case of dentin, hence an increase in cell adhesion on dentin of about 50 % (Fig. 2a).

### Quantification of Osteoclastic Resorption on Bone and Dentin

Resorption activity of osteoclasts was analyzed by determining the following parameters: total resorbed area, average pit size, number of pits per square centimeter, mean pit length, and mean pit width on bone and dentin.

Quantification of the total resorbed area revealed that 0.6 % of the surface was resorbed in bone, whereas 6.7 % of the surface was resorbed on dentin (Fig. 2b), which represents an 11-fold higher osteoclastic resorption activity on dentin compared to bone.

Analyses of the number of pits on the two materials showed that 449 pits/cm² were found on rough bone, whereas on rough dentin we found 3,134 pits/cm² (Fig. 2c).

**Fig. 3:**
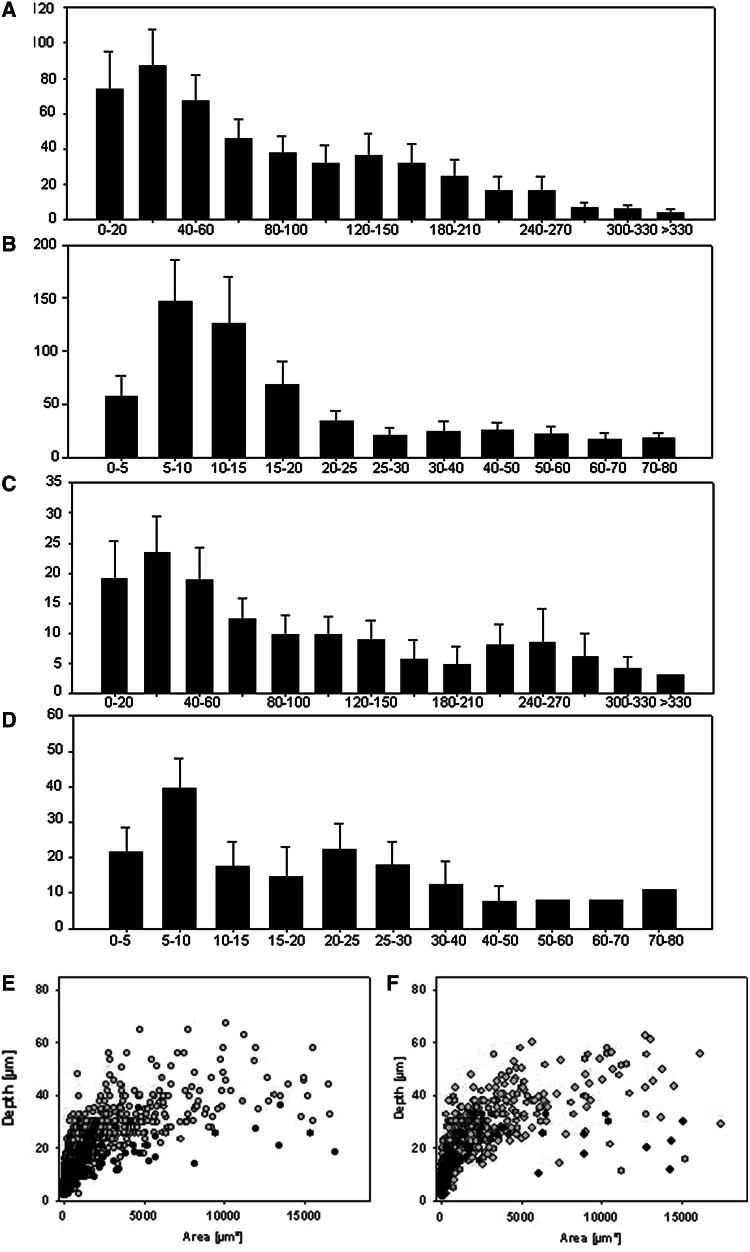
Histograms showing pit length (**a**) and pit width (**b**) distribution on dentin. Histograms showing pit length (**c**) and pit width (**d**) distribution on bone. **a**–**d** *x*-axis represents absolute numbers of pits per category; *y*-axis represents dimensions in micrometers. **a, c** *Bars* 1–14 stand for 0–20, 20–40, 40–60, 60–80, 80–100, 100–120, 120–150, 150–180, 180–210, 210–240, 240–270, 270–300, 300–330, and >300 μm, respectively. **b, d** *Bars* 1–11 stand for 0–5, 5–10, 10–15, 15–20, 20–25, 25–30, 30–40, 40–50, 50–60, 60–70, and 70–80 μm, respectively. *Bars* represent mean ± SEM; *n* = 16 different donors. **e, f** Diagrams showing the depth/area relationship of resorption pits in osteoclast cultures from three different donors from two experiments (**e** experiment 1, **f** experiment 2). Donor 1, *black symbols*; donor 2, *green symbols*; donor 3, *gray symbols*

Interestingly, analyses of average pit size did not show a significant difference between bone and dentin. We found that resorption pits exhibited a size of approximately 580 μm² on rough bone surfaces and of approximately 700 μm² on rough dentin surfaces (Fig. 2d), which reflects the average work performed by each single osteoclast. Detailed analysis of resorption pits revealed a mean pit length of 53 + 6.3 μm for pits/trails on rough bone and a mean pit length of 59 + 6.6 μm for pits/trails on rough dentin. Values for pit width were 9.5 + 1.9 μm on bone and 11 + 1.9 μm on dentin (data obtained from 16 different donors). The histograms in Fig. 3a–d illustrate the distribution of length and width of resorption pits on dentin and bone and both showed similar characteristics on both materials.

We did not find a significant difference between rough and smooth surfaces in the parameters investigated, but we did observe a trend to higher levels on rough surfaces.

These results clearly demonstrate a strong material-dependent effect as well as a material-independent effect on osteoclastic resorption and show that dentin possesses a much higher potential than bone to generate resorbing osteoclasts (percent total resorbed area, number of pits per square centimeter). But once the resorption process itself has started, it takes place quite independently from the material properties (average pit size, mean pit length, mean pit width, and histograms).

### Resorption Pit Depth

We used confocal laser scanning microscopy to measure the depth of resorption pits/trails from osteoclasts cultured on dentin from different blood donors. Interestingly, osteoclasts from all donors showed that pit depth increased to a certain value and then stayed constant, but this maximal pit depth was different for each donor. Reaching the maximal depth, no further increase in pit depth but just an additional increase in area was observed. Figure 3e shows resorption depths of single pits from three different donors; in the case of donor 1, osteoclasts created pits up to a depth of 35 μm, osteoclasts from donor 2 created pits up to a depth of 55 μm, and osteoclasts from donor 3 resorbed pits with a maximum depth of 68 μm. Figure 3f shows a second experiment from these donors, obtained from an independent blood sample, where osteoclasts from donor 1 created pits up to a depth of 32 μm, osteoclasts from donor 2 created pits up to a depth of 59 μm, and osteoclasts from donor 3 resorbed pits with a maximum depth of 62 μm. These similar results indicate that varying pit depth is rather an intrinsic property of the cells than a matter of different culture conditions.

### Microarray Analysis of Genes Expressed in Osteoclasts Cultured on Bone and Dentin

**Fig. 4:**
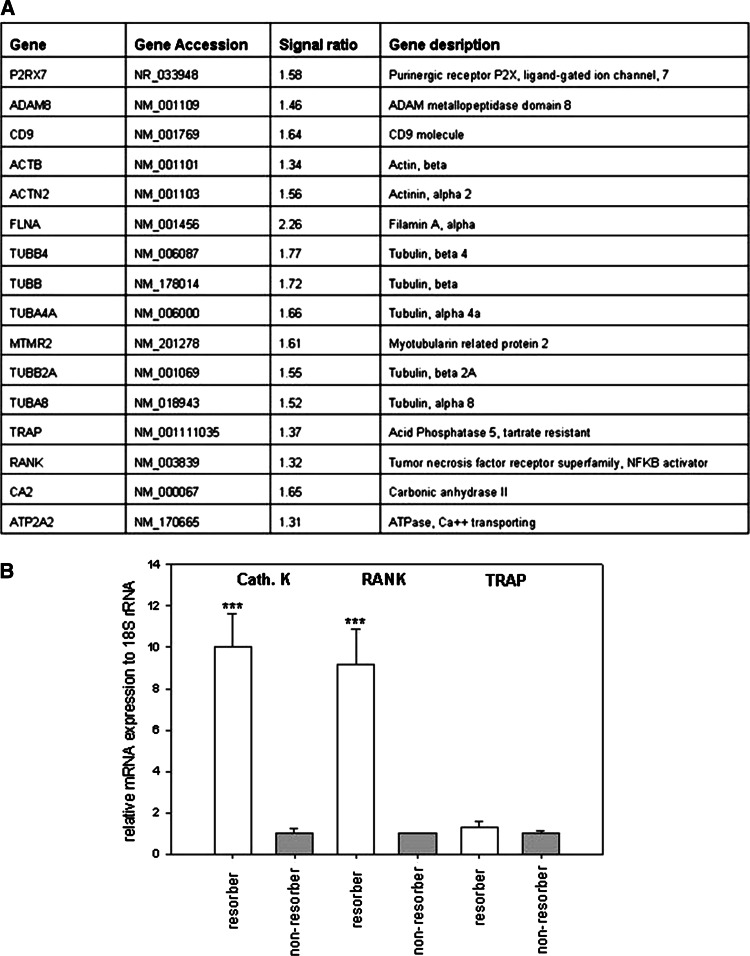
**a** Microarray analyses from osteoclastic cultures on bone and dentin. Signal ratio beyond 1.3 gives the upregulation of genes in cultures on dentin compared to bone. **b** mRNA expression of cathepsin K, RANK, and TRAP in resorbing and nonresorbing osteoclasts cultured on dentin. *Bars* represent mean ± SEM, nonresorbing osteoclasts versus resorbing osteoclasts. Values form nonresorbing osteoclasts were set to 1, and values from resorbing osteoclasts are expressed as *x*-fold. ***p < 0.001

Since preosteoclasts seeded onto a mineralized substrate fuse and form mature resorbing cells [16], we decided to use microarray analysis to determine whether increased resorption on dentin compared to bone was related more to fusion-associated or more to resorption-associated genes. We found that genes related to cell fusion, such as CD9, purinergic receptor (P2RX7), and ADAM 8, and cytoskeleton-related genes like β-actin (ACTB), actinin (ACTN2), filamin A (FLNA), and tubulins (TUB-beta, TUB-alpha, MTMR2) were increased in cells cultured on dentin compared to bone. Furthermore, genes typical for mature, resorbing osteoclasts, e.g., RANK, tartrate-resistant acid phosphatase (TRAP), carbonic anhydrase (CA2), and ATPase (ATP2A2), were also upregulated in osteoclasts cultured on dentin compared to bone (Fig. 4a). Genes associated with the apoptotic pathway (e.g., Fas ligand, Bcl2, BAX, BAD) were not regulated. Caspases 3 and 7 were slightly upregulated, but no other members of the caspase family showed a changed expression level. The regulation of the expression of some typical osteoclastic marker genes (cathepsin K, RANK, and TRAP) was verified in PCR analyses and showed as well an upregulation in resorbing compared to nonresorbing osteoclasts (Fig. 4b). Thus, genes involved in cell fusion and activity were elevated in osteoclasts cultured on dentin compared to bone, which indicates that the increased total resorption on dentin cannot be assigned to one mechanism but may reflect increased fusion as well as resorption activation.

### Resorbing Osteoclasts

**Fig. 5:**
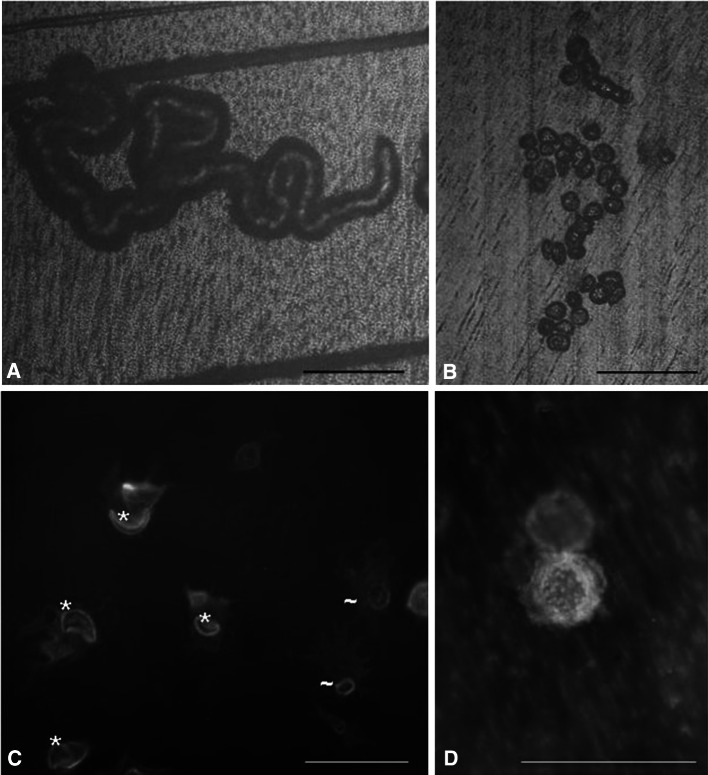
Reflection microscopic image from osteoclastic resorption trail (**a**) and pits (**b**) on a dentin surface. **c** Immunofluorescence staining of actin (*red*) and vitronectin receptor (*green*). Shown are resorbing osteoclasts on dentin, which organize their actin cytoskeleton into a typical belt or ring structure, thus forming the resorbing organelle. *Moon-like or half-elliptical shape, ~round/elliptical shape. **d** A pit-resorbing osteoclast on dentin after immunofluorescence staining. **c, d** *Red* actin staining of the cell, *green* calcein staining of dentin. **a**–**c** *Scale bar* 100 μm, **d** *Scale bar* 50 μM

Two types of resorption traces could be distinguished after osteoclastic resorption. First, single pits, which exhibit a round/elliptical shape, may result from the activity of one single, nonmigrating osteoclast. Second, resorption trails most likely result from the resorption activity of an osteoclast migrating over the surface. Both types of resorption traces display a sharp border between the resorbed area and the rest of the material and were found on dentin as well as on bone (Fig. 5a, b). The resorbing osteoclasts expressed a marker characterizing mature osteoclasts, the vitronectin receptor, and showed the typical F-actin ring within the cell, which represents the resorbing organelle. The resorption organelles were round/elliptical, which were associated with single pits, or exhibited crescent-shaped or half-elliptical shapes, which were associated with osteoclasts forming trails (Fig. 5c).

### 3D Analyses of a Trail-Resorbing Osteoclast

Resorption pits appear under the microscope as round traces. These structures are created by a stationary resorbing osteoclast sealing the whole (round) area, which is going to be resorbed (Fig. 5d).

**Fig. 6:**
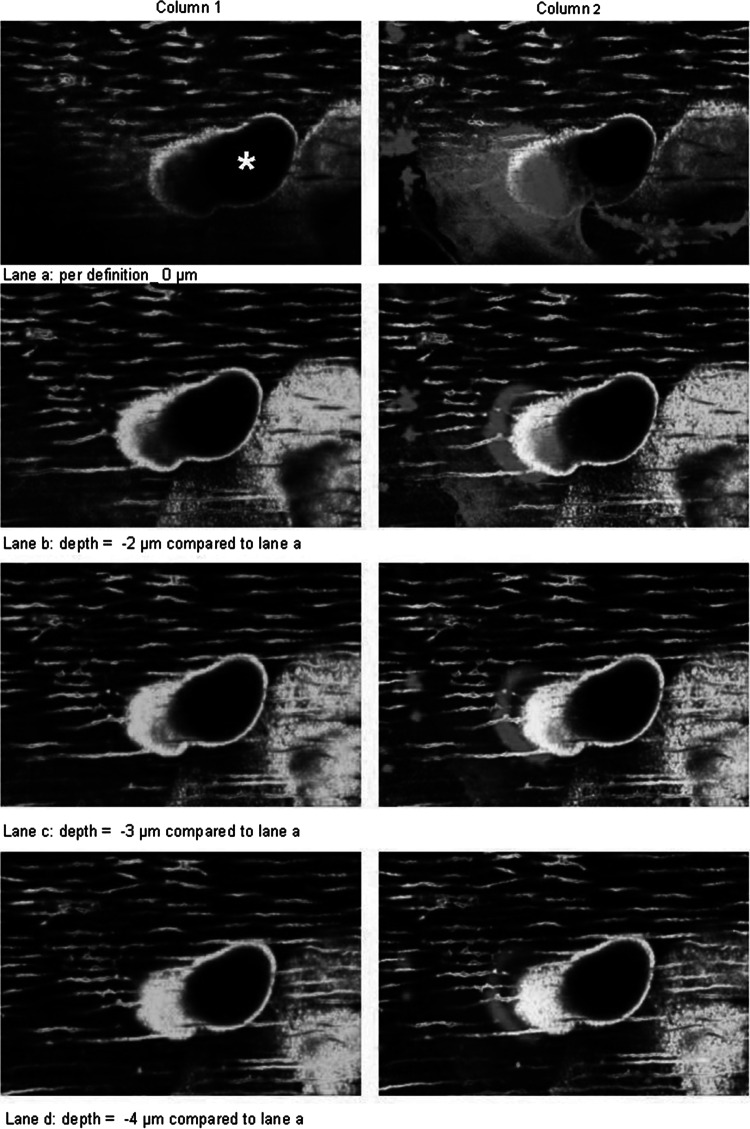
3D imaging of a resorbing osteoclast with confocal laser scanning microscopy. Column 1 = calcein staining of dentin (*green*); column 2 = overlay pictures of actin staining (*red*) and calcein staining (yellow signal = overlay of red and green); *lane a* = depth per definition = 0 μm; depth of *lane b* = 2 μm; depth of *lane c* = 3 μm, depth of *lane d* = 4 μm relative to lane *a*. *Resorption trail

**Fig. 7:**
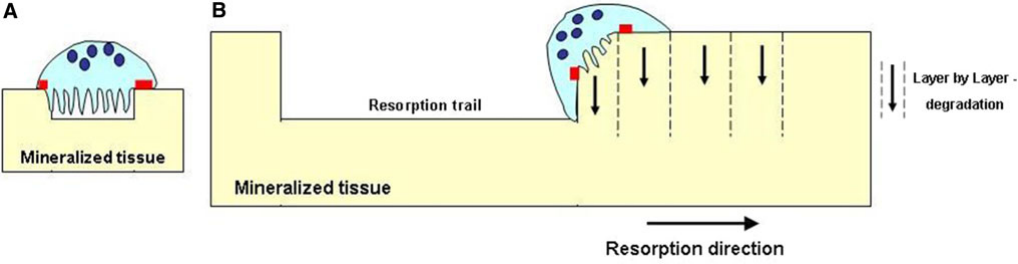
Resorbing osteoclasts. **a** An osteoclast forming a resorption pit as proposed by the literature. **b** Scheme according to our model for osteoclastic trail formation. Osteoclasts degrade the material layer by layer, starting resorption from the top and going in deep. By cyclic repetition of this process a longitudinal resorption trace is formed. *Red bars* mark the actin ring attached to the mineralized matrix

Resorption trails appear in reflected light microscopy as longitudinal resorption traces, suggesting that they are generated by horizontal resorption progression of the osteoclast. To investigate how this resorption process occurs in more detail, we analyzed the 3D architecture of the actin ring of a resorbing osteoclast in an emerging trail on dentin using immunofluorescence staining and confocal laser scanning microscopy. The results clearly show that, in the case of trail formation, one side of the resorption ring, which exhibits a crescent-like structure, attaches to the surface of the material slightly ahead of the matrix border (outside the trail margin). The other side of the actin ring attaches to the wall inside the trail (Fig. 6). Figure 6 gives a series of pictures in *z*-dimension obtained by confocal laser scanning microscopy from the apical part of the resorbing osteoclast into the depth of the emerging resorption trail. In these images, colocalization of the actin staining (red) and calcein staining (green) gives a brilliant yellow color. The emerging resorption trail revealed two parts (Fig. 6, column 1): one part being a “ramp” (green area within the trail margin) and the other part being a “deep hole” (black area within the trail margin), where resorption has already stopped. The attachment of one part of the osteoclast to the surface outside the trail margin is visualized by the red region of the actin ring, slightly ahead in the direction of resorption. The other part of the actin ring attaches to the wall inside the trail (yellow circle-like structure of the actin ring) (Fig. 6, column 2, lane b). From this pattern, the actin ring spans a narrow area and thereby encircles that part of the material which is going to be resorbed next. In our experiments, the horizontal distances between the two walls of crescent-shaped actin rings were approximately 3–21 μm. By going deeper, it is obvious that the actin ring clearly attaches along the “ramp” of the trail (“movement” of the yellow signal in Fig. 6, column 2, lanes b–d), thus forming a hemisphere-like closed resorption organelle. Due to this anatomical configuration of the actin ring, the material is resorbed in the form of a small layer from the surface to the depth. Based on these 3D data, we propose the following model for osteoclastic resorption leading to trail formation: an osteoclast degrades a thin layer of the matrix from the surface to the depth (from the top to the bottom), moves on, and degrades the next thin layer from the surface to the depth (Fig. 7). The numerous repetitions of this cycle (a special sequential combination of vertical material degradation and horizontal movement) leads to the formation of the longitudinal resorption traces seen by microscopy. Thus, the 3D configuration of the resorption organelle only allows a layer-by-layer degradation of the substrate material from the top to the bottom and not a purely horizontal resorption progression of the cell.

## Discussion

The aim of this study was to describe the cellular mechanism of the osteoclastic resorption process in detail and to investigate the resorption behavior of osteoclasts on two widely used mineralized substrates, bone and dentin. Bone is the continuously remodeled mineralized tissue in the human body, and dentin is widely used as an in vitro model substrate to study osteoclastic behavior, very often followed by extrapolation of the data obtained on dentin to osteoclastic behavior on bone. Therefore, we first intended to characterize and compare osteoclastic resorption on these two mineralized materials. Quantification of the resorption behavior revealed, on the one hand, a strong material-dependent effect illustrated by an 11-fold higher resorption on dentin (percent resorbed area) and a sevenfold higher number of pits per square centimeter on dentin compared to bone. This material-dependent effect was also mirrored at the molecular level since genes associated with osteoclast migration, fusion, cytoskeletal organization, and resorption were upregulated in cells cultured on dentin compared to bone. These elevated levels of fusion-related as well as maturation-related osteoclastic genes (cathepsin K and RANK were significantly upregulated, whereas TRAP showed a clear trend for higher expression in resorbing compared to nonresorbing osteoclasts) make it impossible to assign the material-dependent effect to either a pure cell fusion-specific or a pure resorption-specific effect. Unfortunately, we could not study the effect of the substrates on gene expression at the protein level due to the limited amount of cells.

On the other hand, an important material-independent aspect was reflected by a similar average size of the pits on bone and dentin (with slightly higher, but not significantly different, levels on dentin) and comparable mean pit length and width and histogram distribution patterns.

The material chemistry of the substrate seems to have a minor effect on cell adhesion but exerts a major impact on the generation of osteoclasts (number of pits per square centimeter), whereas once the resorption process itself has started, it occurs quite independently of the material properties (average size of pits, mean pit length and width). Interestingly, the resorption does not seem to depend on collagen orientation in the near surface region of the tissue since it appears randomly on osteonal as well as interstitial bone, which definitively exhibit different collagen orientation. Vice versa, bone impairs osteoclastogenesis compared to dentin. Azari et al. [17] described a similar observation, where different mineralized tissues evoked different effects on osteoclast formation and mainly affected their formation rather than their resorption. Nevertheless, the resorption process itself is likely to be the same on bone and dentin. Our observations suggest that dentin is a suitable model substrate for data acquisition and extrapolation to bone when studying general osteoclastic resorption processes. However, addition of pharmacological substances other than M-CSF or RANKL or genetic manipulation of the preosteoclasts may influence or even abolish this observed material-dependent effect. The striking quantitative difference in osteoclastic resorption between bone and dentin is interesting since bone and dentin are very similar in chemical composition as well as structural organization of collagen and mineral crystals at the micrometer and nanometer levels [18, 19]. One possible explanation for the divergence in the observed resorption behavior on both materials may come from a difference in absolute amount of noncollagenous matrix proteins, e.g., osteopontin. This protein is present in various concentrations in diverse bone types and is speculated to influence cell behavior [17]. In addition, osteocytic proteins, which are present in bone but are missing in dentin, may act on osteoclasts in the bone remodeling processes [20–23]. Removal of organic components and osteocytic proteins from bone matrix was followed by an increased osteoclastic resorption in vitro [14]. Different osteoclastic resorption activity has also been described for numerous artificial substrates, e.g., hydroxyapatite, β-tricalcium phosphate, calcium phosphate, calcite, and carbonated apatite, including variation of cell adhesion rate on those materials, which we observed as well between bone and dentin [24–27]. Osteoclastic activity is even influenced by extracts from natural mineralized substrates [28, 29]. However, it is not understood why some substrates favor osteoclast adhesion and resorption while others do not. Beyond those prominent material-dependent effects, we did not find a significant roughness-dependent effect in the micrometer range we offered to cells (just a tendency to higher levels on rough surfaces). Geblinger et al. [30–32] reported an increased half-life of the actin rings of resorbing RAW 264.7 osteoclast-like cells on rough artificial calcium substrates compared to smooth ones (roughness in the nanometer range), which could explain our observed tendency for higher resorption on the rough surfaces, as well as a very high dynamics of the sealing-zone being locally regulated by surface roughness. However, topography-dependent higher resorption on rougher surfaces is described in the literature in osteoclast-like cell lines and osteoclasts generated from bone marrow [33–36]. Nevertheless, the effects in cultures of primary osteoclasts may be due to the presence of osteoblastic cells or stromal cells, which are well known to strongly affect osteoclastic behavior [34]. Our osteoclast cultures were generated from peripheral blood; thus, they did not contain contaminating stromal cell types. This allowed examination of the direct behavior of osteoclasts and not the indirect effects caused by the communication between osteoclasts and other cell types. Besides the fact that osteoclasts are supposed to be significantly influenced by interactive signals from other cells, our data show that osteoclasts can, to a certain degree, act independently from mesenchymal cell types. The material itself as well as the surface topography of the underlying substrate have the potential to directly guide the resorption behavior of osteoclasts (as reflected by the size and number of resorption lacunae and the resorbed area). In the resorption process, actin ring formation is a prerequisite for an osteoclast to resorb; and in the case of bone as well as dentin, this process results in the same typical resorption traces, pits and trails, visible under reflected light microscopy. Resorption pits correspond to “reticulate patch resorption” patterns, and resorption trails correspond to the “longitudinal” patterns, categorized by Gentzsch et al. [37]. These resorption traces were also found in bone of femoral heads in vivo [37]. The 3D analyses of the resorption depth in our cultures showed that osteoclasts resorb up to certain maximum depth. After reaching this depth, osteoclasts continue their resorption to further increase area but not depth. This superficial resorption behavior of osteoclasts corresponds to the “lacunar perforation type” described in vivo [37]. Surprisingly, the resorption potential of the osteoclasts, generated from peripheral blood, strongly varied between the different donors; but nevertheless, it seems to be an intrinsic potential of the osteoclasts themselves. This was reflected by the varying general resorption potential on the substrate surfaces, reflected by quite large error bars in the histograms, as well by the maximal depth of the resorption pits and pit volume. Such a high variability in the resorption potential of osteoclasts was also observed by other authors [11, 38]. We speculate that the individual, indefinable condition of each donor, probably reflected in the serum parameters but not further qualified in the case of our donors, played a significant role in this phenomenon since our donors were healthy individuals aged 19–55 years.

The resorption traces formed by osteoclasts seem to be identical not only between bone and dentin but also to other mineralized substrates since osteoclasts always form either pits or trails or fusing trails. Beyond that, it is intriguing that on all materials and in osteoclasts from all species used in in vitro studies, the same actin ring formation patterns were observed [10–13]. In the case of single pit formation, the resorption mechanism is known in the literature, which describes that the resorption direction is exclusively oriented to depth and the actin ring forms a circular or elliptical shape, enclosing the material in one plane [9]. Such a single pit may function as the initial step for trail formation. The other shapes of actin rings observed, namely, crescent-shaped and half-elliptical, were associated with emerging resorption trails. Saltel et al. [39] also observed round or crescent-shaped actin rings in osteoclasts seeded on hydroxyapatite or dentin. Due to lacking information about the trail-forming process, we sought to elucidate the principal mechanism of how such a resorption trail is generated by an osteoclast. A resorption trail is most probably produced by one osteoclast that migrates over the surface during the resorption process, whereas pits result from a noncontinuous process [40]. Based on the picture of a resorption trail visible under the microscope, one could speculate that such a longitudinal resorption trace may come from an osteoclast that resorbs to a certain depth and then continues resorption by a simple horizontal progression along the surface in a “worm-like style.” Under confocal laser scanning microscopy we investigated actin rings which appeared as half-elliptical or crescent-shaped structures and were directly associated with an emerging trail. A limitation of this study is that investigations were done on fixed cells, thus catching just one moment in the resorption process and not revealing dynamics in living cells. Nevertheless, based on the observed 3D configuration of the actin ring due to trail architecture in case of resorbing osteoclasts we developed a model to explain how the resorption process leads to trail formation: during trail formation, one side of the actin ring is attached to the surface of the material, namely, outside the existing trail margin in the direction of resorption. The other side of the ring attaches to the inner margin of the trail and reaches down along the material wall; thus, the closed resorption organelle encircles the region that is going to be resorbed next. This 3D architecture of the resorption organelle allows the resorption of a thin layer of material from the surface to a depth (from top to bottom) which may lie, according to our analyses, in the range of 3–21 μm. After reaching a certain pit depth, the cell stops resorption and the actin ring attaches again one step onward in the direction of resorption on the material surface, again followed by resorption of the material underneath. By a cyclic repetition of this procedure, always starting resorption from the top and proceeding down to the depth, the cell degrades the material layer by layer. Thus, the anatomical configuration of this kind of resorption organelle does not allow a simple horizontal resorption movement in a “worm-like style” but only a layer-by-layer degradation of the material from the top to the bottom, at the end appearing as a longitudinal trace under the microscope. In our cultures we found only trail-resorbing osteoclasts exhibiting the described features. Nevertheless, we cannot exclude that, in general, there may be vertically resorbing osteoclasts as well.

## Conclusion

The formation of resorption trails by osteoclasts occurs by an orchestrated combination of horizontal cell movement and vertical degradation of the material in thin layers.

Dentin is an appropriate substrate for studying osteoclast formation since resorption runs independently of the material (and is comparable on bone and dentin), once it has started. It is the initiation of resorption which seems to be material-dependent and is different on bone and dentin. Thus, only material-related conclusions extrapolated from dentin to bone (e.g., osteoclast formation kinetics) are not reliable. Pharmacological substances or genetic manipulation of cells may abolish this material-dependent effect.
